# Camrelizumab, an Anti‐PD‐1 Monoclonal Antibody, Plus Carboplatin and Nab‐Paclitaxel as First‐Line Setting for Extensive‐Stage Small‐Cell Lung Cancer: A Phase 2 Trial and Biomarker Analysis

**DOI:** 10.1002/mco2.70300

**Published:** 2025-07-27

**Authors:** Jia Yu, Deyu Cai, Sha Zhao, Lei Wang, Xinlong Zheng, Anwen Xiong, Qi Wang, Bin Chen, Menghang Yang, Wei Li, Yan Wang, Jiajia Yuan, Changhong Zhao, Wei Shi, Caicun Zhou, Luonan Chen, Tao Jiang, Xiaohui Chen, Shengxiang Ren

**Affiliations:** ^1^ Department of Medical Oncology Shanghai Pulmonary Hospital, School of Medicine, Tongji University Shanghai People's Republic of China; ^2^ Key Laboratory of Systems Biology Shanghai Institute of Biochemistry and Cell Biology Center for Excellence in Molecular Cell Science Chinese Academy of Sciences Shanghai People's Republic of China; ^3^ School of Life Science and Technology ShanghaiTech University Shanghai People's Republic of China; ^4^ Department of Thoracic Surgery Clinical Oncology School of Fujian Medical University Affiliated to Fujian Cancer Hospital Fuzhou People's Republic of China; ^5^ Interdisciplinary Institute of Medical Engineering of Fuzhou University Fuzhou People's Republic of China; ^6^ Jiangsu Hengrui Pharmaceuticals Co. Ltd. Shanghai People's Republic of China; ^7^ School of Mathematical Sciences and School of AI Shanghai Jiao Tong University Shanghai People's Republic of China; ^8^ Graduate School of Fujian Medical University Fuzhou People's Republic of China

**Keywords:** biomarker, camrelizumab, ES‐SCLC, immunotherapy, nab‐paclitaxel

## Abstract

This study aimed to investigate the efficacy, safety, and predictors of camrelizumab combined with carboplatin and nab‐paclitaxel as first‐line setting for patients with extensive‐stage small‐cell lung cancer (ES‐SCLC). Camrelizumab plus carboplatin and nab‐paclitaxel were administrated every 3 weeks for four to six cycles, followed by maintenance camrelizumab until intolerable toxicity or disease progression. The primary endpoint was 6‐month progression‐free survival (PFS) rate and secondary endpoints were objective response rate (ORR), disease control rate (DCR), PFS, overall survival (OS), and safety. We conducted the whole‐exome and transcriptomic sequencing on available tumor samples to explore the potential predictive biomarkers. A total of 60 patients were included. Primary endpoint was met with 6‐month PFS rate of 52.2%. The median PFS and OS were 7.1 and 18.1 months, respectively. The confirmed ORR and DCR were 73.3% and 93.3%, respectively. No unexpected adverse events were observed. Exploratory analysis showed that *MUC17* alterations or high *NEUROG1* expression were correlated with markedly shorter PFS and OS. Deeper investigation of transcriptomic data reveals two subsets with distinct immune features and therapeutic vulnerabilities. Collectively, this trial suggested that camrelizumab plus carboplatin and nab‐paclitaxel might be an alternative first‐line setting for ES‐SCLC. Integration of multiomic data could highlight the complex mechanisms underlying chemo‐immunotherapy responses.

## Graphical abstract

This study firstly provides evidence for the efficacy, safety, and potential predictive biomarkers of camrelizumab in combination with carboplatin and nab‐paclitaxel as first‐line treatment for patients with extensive‐stage small‐cell lung cancer, suggesting that this regimen could serve as an alternative first‐line setting for these patients.

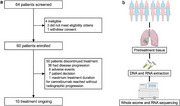

## 1 Introduction

Small‐cell lung cancer (SCLC) is one of the common histological types of lung cancers with high metastatic potential and dismal prognosis [1, 2, 3, 4, 5]. It is characterized by early distant metastasis, a short tumor doubling time and low 5‐year survival rate [6, 7]. Approximately 75% of patients with SCLC are initially diagnosed with extensive‐stage SCLC (ES‐SCLC) [8]. During the past decades, platinum‐based chemotherapy (with irinotecan or etoposide) has been the standard of care for ES‐SCLC [9, 10]. The use of irinotecan plus platinum is restricted in clinical practice due to the relatively poor absolute survival benefit and toxicity profile [11, 12, 13]. Therefore, the first‐line chemotherapeutic drugs available for patients with ES‐SCLC are relatively limited.

Recently, several elegant Phase 3 trials, including IMpower133, CASPIAN, ASTRUM‐005, CAPSTONE‐1, RATIONALE‐312, and EXTENTORCH evaluated blockade of PD‐1 and PD‐L1 interactions plus etoposide and platinum as first‐line treatment for ES‐SCLC and reported the consistently prolonged survival benefit than control groups [14, 15, 16, 17, 18, 19, 20], which have become the new standard first‐line setting for ES‐SCLC. Although most of PD‐1/PD‐L1 antibodies need only 1‐day infusion, traditional etoposide infusion still needs 3 days. Considering the inconvenience and limited options of current first‐line choice, there is urgent need to investigate alternative combinatorial treatment regimens for these patients.

Nab‐paclitaxel is an albumin‐bound, solvent‐free, formulation of paclitaxel which could minimize the toxic effect of traditional paclitaxel treatment [21]. It has showed promising antitumor efficacy in advanced breast, pancreatic cancer and non‐small‐cell lung cancer (NSCLC) [22, 23]. Our previous Phase 2 trial observed the promising efficacy and acceptable toxicity of nab‐paclitaxel as second or later‐line treatment for ES‐SCLC [24]. In this trial, we aimed to investigate the efficacy and safety of camrelizumab combined with nab‐paclitaxel and carboplatin as first‐line treatment for patients with ES‐SCLC. In addition, we also performed the whole exome and transcriptomic sequencing on high‐quality pretreatment tumor tissues to investigate the potential predictive biomarkers in tumor specimens that may be indicative of clinical efficacy.

## 2 Results

### 2.1 Patient Characteristics

**FIGURE 1:**
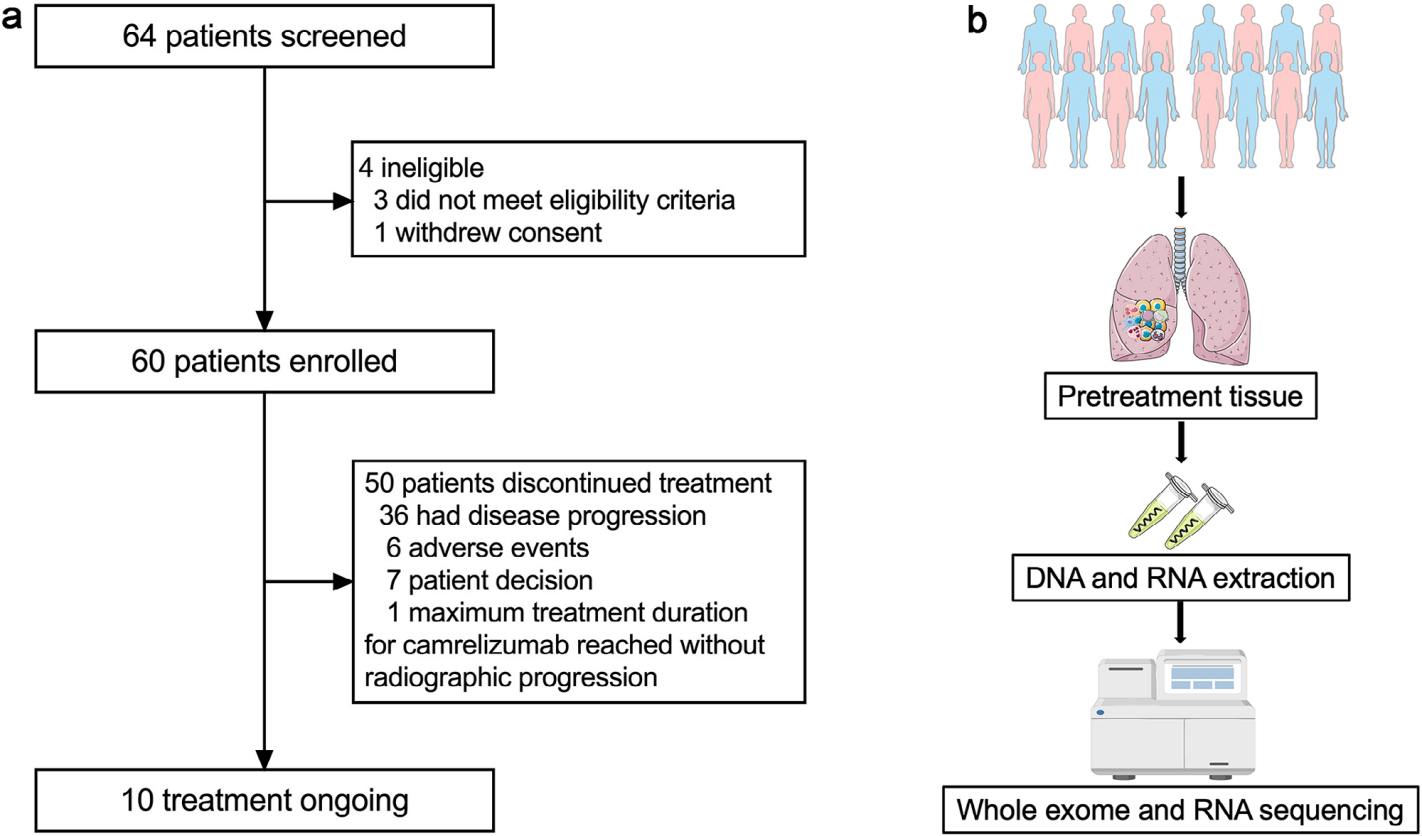
Flow diagram of this study. (a) Patients’ selection. (b) Sample collection and biomarker analysis.

Between March 31, 2021 and December 14, 2022, 60 patients with ES‐SCLC were included. All patients were treated with ≥ 1 dose of study treatment and included in the final analyses (Figure 1a). A total of 38 cases with high‐quality baseline tumor samples were included in the biomarker analyses (Figure 1b). The median follow‐up duration was 19.3 months (95% CI: 16.2–22.8 months; Figure S1). Ten (16.7%) patients were still receiving the study treatment. A total of 50 patients discontinued the study treatment because of PD (*n* = 36, 60%), AEs (*n* = 6, 10.0%), patient decision (*n* = 7, 11.7%), or end of treatment per study design (*n* = 1, 1.7%). The baseline features are listed in Table 1. The median age was 65 years (range, 38–74). Most patients were male (50 [83.3%]), former smokers (40 [66.7%]), with an Eastern Cooperative Oncology Group (ECOG) performance status of 1 (60 [100%]), and had stage IV disease at diagnosis (52 [86.7%]). Seven (11.7%) patients had central nervous system (CNS) metastases, 10 (16.7%) patients had liver metastases, and 21 (35.0%) patients had elevated lactate dehydrogenase.

**TABLE 1:** Baseline characteristics of all included patients.

Characteristics	All patients (*n* = 60)
Age, years, median (range)	64.5 (38–74)
< 65 years, *n* (%)	34 (56.7)
≥ 65 years, *n* (%)	26 (43.3)
Male, *n* (%)	50 (83.3)
ECOG performance status, *n* (%)	
0	0 (0)
1	60 (100)
Disease stage, *n* (%)	
III	8 (13.3)
IV	52 (86.7)
Smoking status, *n* (%)	
Current or former smoker	40 (66.7)
Never smoked	20 (33.3)
Brain metastases, *n* (%)	
Yes	7 (11.7)
No	53 (88.3)
Liver metastases, *n* (%)	
Yes	10 (16.7)
No	50 (83.3)
Lactate dehydrogenase at enrollment	
≤ ULN	39 (65.0)
> ULN	21 (35.0)

Abbreviations: ECOG, Eastern Cooperative Oncology Group; ULN, upper normal limit.

### 2.2 Efficacy

**FIGURE 2:**
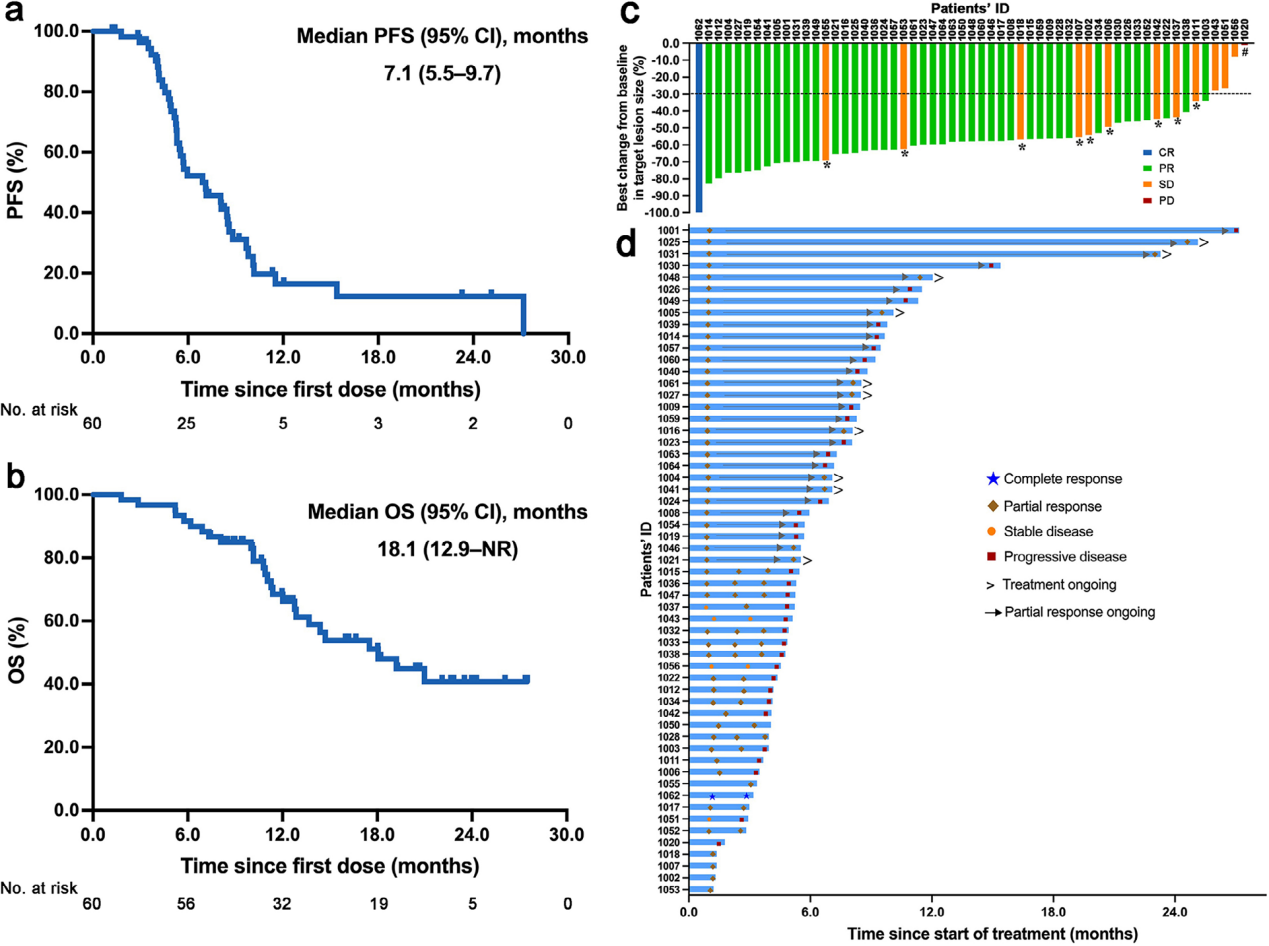
Treatment outcomes of all eligible patients. (a) Kaplan–Meier curves for PFS in all patients (median PFS: 7.1 months, 95% CI: 5.5–9.7 months). (b) Kaplan–Meier curves for OS in all patients (median OS: 18.1 months, 95% CI: 12.9 months–not reached). (c) Best percentage change from baseline in target lesion. (d) Duration of response; the asterisks indicated unconfirmed responses; the pound key indicated this patient had disease progression due to new target lesion at assessment. CI, confidence interval; NR, not reached; OS, overall survival; PFS, progression‐free survival.

At data cutoff, 27 (45%) of them had died. According to the assessment of progression‐free survival (PFS), 36 (60.0%) patients had disease progression (PD) or died. The 6‐month PFS rate was 52.2% (95% CI: 39.8%–68.5%) and the median PFS was 7.1 months (95% CI: 5.5–9.7 months; Figure 2a). The median overall survival (OS) was 18.1 months (95% CI: 12.9–not reached [NR] months) and the 1‐year OS rate was 68.4% (95% CI: 56.8%–82.3%) (Figure 2b). Figure 2c summarized the best change in the sum of diameters of target lesion from baseline. Fifty‐six (93.3%) patients had decreased total tumor burden. One (1.7%) patient achieved complete response (CR), 43 (71.7%) patients achieved partial response (PR) as their best response, 12 (20.0%) patients had stable disease (SD), and 1 (1.7%) patient had PD and the overall responses of 3 (5%) patients were not evaluable (Table S1). The confirmed objective response rate (ORR) was 73.3% (95% CI: 60.3%–83.9%) and disease control rate (DCR) was 93.3% (95% CI: 83.8%–98.2%). The onset of response was fast (Figure 2d), with a median time to response of 1.4 months (range, 0.7–2.7). The median duration of response (DoR) was 6.7 months (95% CI: 5.1–8.2 months; Figure S2a). The efficacy data from 11 patients received etoposide and platinum as the second‐line treatment after PD were collected retrospectively. Three patients achieved PR, 7 patients achieved SD, and 1 patient had PD. The median PFS was 5.7 months (Figure S2b).

### 2.3 Safety

All patients were eligible for safety analysis. The median cycle number of camrelizumab treatment was 7.9 (range, 1–33), and the median cycle number of chemotherapy exposure was 4.1 (range, 1–6). Treatment‐related adverse events (TRAEs) occurred in 59 (98.3%) patients. Grade ≥ 3 TRAEs occurred in 34 (56.7%) patients (Table 2). The most commonly reported TRAEs were anemia (83.3%), platelet count decreased (60%), neutrophil count decreased (58.3%), white blood cell count decreased (50%), and alopecia (43.3%). The most common grade ≥ 3 adverse events (AEs) were decreased of neutrophil count (31.7%), white blood cell count (15.0%), platelet count (13.3%), and increased of gamma‐glutamyltransferase (10.0%) and blood bilirubin (10.0%). Reactive cutaneous capillary endothelial proliferation was observed in 15 (25.0%) patients, and all were Grade 1 or 2. Treatment‐related severe AEs occurred in 21 (35.0%) patients, with immune‐mediated hepatitis (*n* = 3 [5.0%]) and platelet count decreased (*n* = 3 [5.0%]) as the most common AEs (Table S2). TRAEs leading to treatment discontinuation occurred in 6 (10.0%) patients. Fatal AEs possibly related to study treatment were occurred in one (1.7%) patient (suspected checkpoint inhibitor related hepatitis and nephritis). No unexpected AEs were observed.

**TABLE 2:** Summary of treatment‐related adverse events for all included patients.

Adverse events	All patients, *n* (%)	
	Any grade	≥ Grade 3
Any TRAE	59 (98.3)	34 (56.7)
Hematological toxicities		
Anemia	50 (83.3)	3 (5.0)
Platelet count decreased	36 (60.0)	8 (13.3)
Neutrophil count decreased	35 (58.3)	19 (31.7)
White blood cell count decreased	32 (50.0)	9 (15.0)
Nonhematological toxicities		
Alopecia	26 (43.3)	0 (0.0)
Aspartate aminotransferase increased	23 (38.3)	5 (8.3)
Alanine aminotransferase increased	20 (33.3)	5 (8.3)
Gamma‐glutamyltransferase increased	16 (26.7)	6 (10.0)
RCCEP	15 (25.0)	0 (0.0)
Blood alkaline phosphatase increased	14 (23.3)	1 (1.7)
Hypesthesia	13 (21.7)	1 (1.7)
Asthenia	13 (21.7)	0 (0.0)
Occult blood positive	12 (20.0)	0 (0.0)
Nausea	11 (18.3)	0 (0.0)
Vomiting	11 (18.3)	0 (0.0)
Blood creatinine increased	11 (18.3)	1 (1.7)
Blood bilirubin increased	10 (16.7)	6 (10.0)
Rash	10 (16.7)	1 (1.7)
Constipation	8 (13.3)	0 (0.0)
Protein urine present	8 (13.3)	0 (0.0)
Hyperglycemia	6 (10.0)	1 (1.7)
Hyperthyroidism	5 (8.3)	0 (0.0)
Hypothyroidism	4 (6.7)	0 (0.0)
Infusion reaction	2 (3.3)	0 (0.0)

Abbreviations: TRAE, treatment‐related adverse event; RCCEP, reactive cutaneous capillary endothelial proliferation.

### 2.4 Biomarker Analysis

**FIGURE 3:**
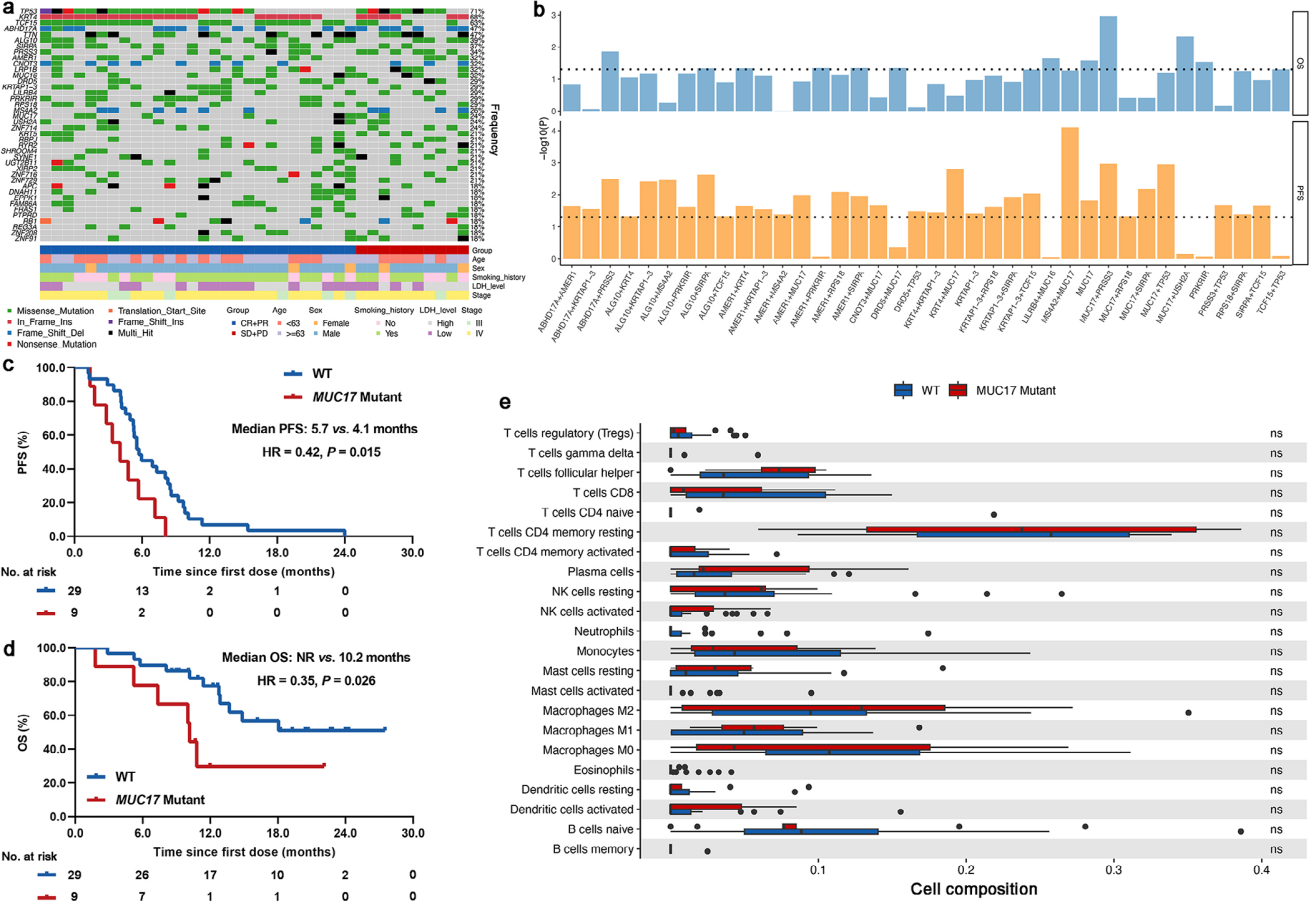
Whole‐exome sequencing data analysis. (a) Mutational landscape of all patients. (b) *p* value of top 20 frequent gene alterations or their combinations associated with treatment outcomes. (c) PFS comparison between patients with *MUC17* alterations and wild type (median PFS: 4.1 vs. 5.7 months, *p* = 0.015). (d) OS comparison between patients with *MUC17* alterations and wild type (median OS: 10.2 vs. NR months, *p* = 0.026). (e) Comparison of immune infiltrates estimated by CIBERSORT between patients with *MUC17* alterations and wild type. HR, hazard ratio; NR, not reached; OS, overall survival; PFS, progression‐free survival; WT, wild type.

Biomarker analysis was limited to SCLC patients with available tumor biopsies. After quality assessment, 41 tissue samples were sequenced. Three of them were excluded due to low‐quality sequencing data. Herein, 38 cases were included. The baseline parameters were balanced between the biomarker‐evaluable population (BEP) and the intention‐to‐treat (ITT) population (Table S3). The efficacy was also similar to the ITT population (Figure S3). First, we pictured the mutational landscape of included cases and investigated their associations with treatment outcomes (Figure 3a). The most common genetic alteration was *TP53* alterations (71%). *RB1* alterations were observed in 18% of patients. Given the small size of BEP, we set a loose criterion (*p* ≤ 0.05) to select genes or their combinations with potential predictive value and the minimum number of samples for each group should be more than 20% of whole population. Following these criteria, we observed the prediction model would have reliable performance when the number of genes reaches two and several genes or their combinations were associated with treatment outcomes (Figure 3b). The double alterations of *MUC17* and *PRSS3* showed the best predictive value after screening (Figure 3b). Interestingly, *MUC17* could be considered as a potential predictive biomarker, while *PRSS3* alone cannot (Figure 3b). Patients with *MUC17* alterations had significantly shorter PFS (median PFS: 4.1 vs. 5.7 months, *p* = 0.015; Figure 3c) and OS (median OS: 10.2 vs. NR months, *p* = 0.026; Figure 3d) than those without *MUC17* alterations. Nevertheless, immune infiltration analysis using CIBERSORT showed analogous immune infiltration between *MUC17* alterations and wild type (Figure 3e). Then, we evaluated the predictive significance of tumor mutational burden (TMB) and found that patients with high TMB (> median) had similar PFS (*p* = 0.129; Figure S4a) and OS (*p* = 0.434; Figure S4b) to those with low TMB (≤ median).

**FIGURE 4:**
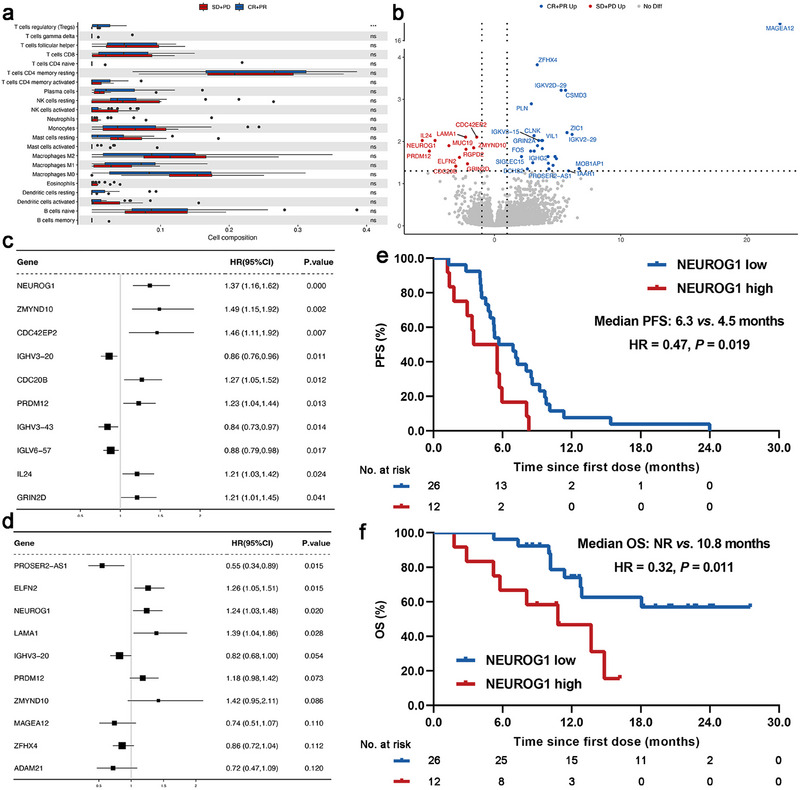
Whole transcriptomic sequencing data analysis. (a) Comparison of immune infiltrates estimated by CIBERSORT between CR+PR and SD+PD groups. (b) Volcano plot of 17 upregulated and 11 downregulated differentially expressed genes between CR+PR and SD+PD groups, all of them labeled the gene names. (c) Forest plots depicting the significance of dramatically differentially expressed genes in predicting PFS. (d) Forest plots depicting the significance of dramatically differentially expressed genes in predicting OS. (e) PFS comparison between patients with high and low *NEUROG1* expression (median PFS: 6.3 vs. 4.5 months, *p* = 0.019). (f) OS comparison between patients with high and low *NEUROG1* expression (median OS: NR vs. 10.8 months, *p* = 0.011). CI, confidence interval; HR, hazard ratio; NR, not reached; OS, overall survival; PFS, progression‐free survival.

To survey the features of immune infiltration corresponding to distinct treatment outcomes, we deconvoluted the transcriptomic sequencing data from 38 samples. In general, most of the immune cell's infiltrations were analogous between CR+PR and SD+PD groups (Figure 4a). Only regulatory T cell infiltration was markedly different between two groups (*p* < 0.0001; Figure 4a). Next, we surveyed the differentially expressed genes (DEGs) corresponding to different therapeutic outcomes. The similarity among samples is relatively strong (Figure S5). Only 38 DEGs were identified between two groups (*p* ≤ 0.05, Log_2_ Fold Change ≥ 2), including 27 upregulated and 11 downregulated genes (Figure 4b). To further demystify the biological pathway alterations, we conducted Gene Ontology (GO) enrichment analyses and found that these DEGs prominently displayed immune‐related functions including antigen binding and immunoglobulin production (Figure S6). We listed the top 10 genes correlated with PFS and OS according to *p* values of the univariate analyses (Figure 4c,d). Among them, only *NEUROG1* expression was markedly correlated with both PFS and OS. Using the median expression level of this study population as the cutoff, patients with low *NEUROG1* expression had markedly superior PFS (median PFS: 6.3 vs. 4.5 months, *p* = 0.019; Figure 4e) and OS (median OS: NR vs. 10.8 months, *p* = 0.011; Figure 4f) than those with high *NEUROG1* expression. Moreover, patients with low *NEUROG1* expression had dramatically higher fractions of activated dendritic cells than those with high *NEUROG1* expression (Figure S7).

**FIGURE 5:**
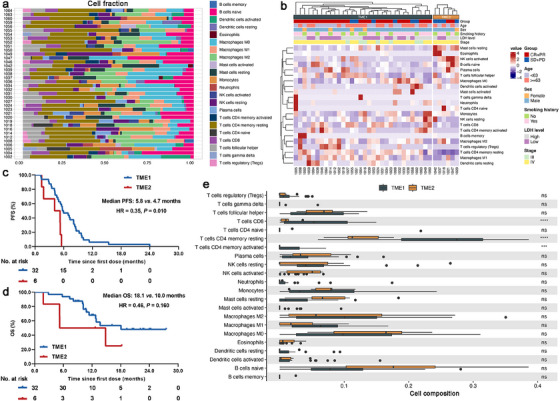
De novo non‐negative matrix factorization (NMF) transcriptomic sequencing data and their associated treatment outcomes. (a) Composition of 22 immune cells in each sample. (b) Two clusters were well‐defined (named TME1 and TME2) by using NMF. (c) PFS comparison between patients in TME1 and TME2 (median PFS: 5.8 vs. 4.7 months, *p* = 0.010). (d) OS comparison between patients in TME1 and TME2 (median OS: 18.1 vs. 10.0 months, *p* = 0.160). (e) Comparison of immune infiltrates estimated by CIBERSORT between TME1 and TME2. NR, not reached; OS, overall survival; PFS, progression‐free survival.

We also applied de novo non‐negative matrix factorization (NMF) to raw transcriptomic data of 38 ES‐SCLC patients received camrelizumab plus nab‐paclitaxel and carboplatin based on the immune cell fractions (Figure 5a). We observed the current set could be divided into two well‐defined clusters (named TME1 and TME2; Figure 5b). TME1 was the major subgroup containing 32 cases and TME2 only had 6 cases. We next investigated the correlation between distinct NMF subsets and treatment outcomes. TME1 had significantly higher rate of responders (CR+PR) than TME2 (ORR: 81.3% vs. 33.3%, *p* = 0.052). Of note, patients in TME1 had dramatically longer PFS (median PFS: 5.8 vs. 4.7 months, *p* = 0.010; Figure 5c) than those in TME2. TME1 also had prolonged OS (median OS: 18.1 vs. 10.0 months, *p* = 0.160; Figure 5d) than TME2 but it did not reach the statistical significance mainly due to the small sample size. We then compared specific immune infiltration features of the two NMF subsets and found that TME1 showed markedly elevated infiltration abundance of CD8+ T cells and memory CD4+ T cells than TME2 (Figure 5e).

## 3 Discussion

Currently, PD‐1 blockade plus chemotherapy has shifted the treatment landscape and become the standard of care for patients with previously untreated ES‐SCLC [25, 26]. Nevertheless, chemotherapy is still the treatment backbone for ES‐SCLC. The first‐line therapeutic options were limited and etoposide is the only clinical choice with 3‐day infusion. Although the initial objective response is high, relapse is rapid and inevitable, which mainly resulted in the poor prognosis of ES‐SCLC [27]. Therefore, development of alternative chemotherapeutic agents with high efficacy, low toxicity and convenient clinical application is urgently needed.

To our knowledge, this trial firstly investigated camrelizumab plus carboplatin and nab‐paclitaxel in patients with untreated ES‐SCLC. Our findings suggest that camrelizumab in combination with carboplatin and nab‐paclitaxel resulted in a favorable PFS and OS in ES‐SCLC. The 6‐month PFS rate was 52.2%. The median PFS and OS were 7.1 and 18.1 months, respectively. This data was noninferior to the reported data in recent published Phase III trials of PD‐1 blockade plus etoposide and platinum as first‐line treatment for ES‐SCLC (e.g., median PFS and OS were 5.2 and 12.3 months in IMpower 133, 5.1 and 12.9 months in CASPIAN, 5.8 and 15.3 months CAPSTONE‐1, 5.8 and 15.8 months in ASTRUM‐005, 4.8 and 15.5 months in RATIONALE‐312, and 5.8 and 14.6 months in EXTENTORCH) [14, 15, 16, 18, 19, 20]. The AEs were manageable and no new safety signals occurred, which was similar to these published Phase III trials. Paclitaxel plus carboplatin or cisplatin as first‐line treatment have shown promising efficacy for advanced NSCLC [28, 29]. The long‐term efficacy of carboplatin and paclitaxel was comparable with etoposide plus cisplatin in ES‐SCLC [30, 31]. Previously, we reported a Phase 2 trial that nab‐paclitaxel showed the promising efficacy and acceptable toxicity as second or later‐line setting in ES‐SCLC [24]. In this study, we further found that camrelizumab combined with nab‐paclitaxel and carboplatin achieved comparable short‐term and long‐term efficacy in contrast to previous reports providing a potential alternative first‐line chemotherapy for ES‐SCLC. Moreover, 17 patients received platinum and etoposide as the second‐line treatment after PD in this study. The efficacy data from 11 patients were collected. The ORR was 27.3% and the median PFS was 5.7 months, indicating that patients progressed after first‐line treatment of immunotherapy plus carboplatin and nab‐paclitaxel could also benefit from platinum and etoposide as subsequent therapy. This might also contribute to the long OS. Collectively, these findings reveal that camrelizumab plus carboplatin and nab‐paclitaxel are an alternative choice in untreated ES‐SCLC.

The safety profiles of this regimen were similar to previous reports of component monotherapies [32, 33]. The most common grade ≥ 3 TRAEs were hematological toxicities. This might be attributed to the higher susceptibility to hematological AEs from chemotherapy in Asian population [34]. In addition, the overall physical condition was worse in our study as all the patients had an ECOG performance status of 1 at baseline [35]. The hematological AEs were managed effectively with standard care. The hepatic function abnormalities might be correlated with camrelizumab or chemotherapy [28, 33]. Of note, RCCEP occurred in 15 (25%) patients, with no grade ≥ 3 AEs reported. Overall, the incidence and severity of TRAEs were acceptable.

Biomarker analysis reported that *MUC17* alterations and *NEUROG1* expression were correlated with treatment outcomes. *MUC17*, encoded a membrane‐bound mucin, played an antiadhesive role in cancer cells that lose their apical/basal polarization and has been reported as a potential negative prognostic biomarker for several solid tumors including breast, glioma, gastric, colon, and biliary tract cancers [36, 37, 38, 39, 40]. Although *MUC17* alterations were correlated with poor clinical outcomes in this study, we cannot rule out the possibility that it was a generally prognostic factor due to the highly similar immune infiltration features between *MUC17* alterations and wild type. *NEUROG1*, as a proneural gene, played a vital role in the initiation of neuronal differentiation. Previous studies focused on the biological role of its methylation on different cancers [41]. The current study firstly reported that high *NEUROG1* expression was correlated with inferior treatment outcomes. Immune deconvolution analysis showed that tumors with low *NEUROG1* expression had a higher fraction of activated dendritic cells than those with high *NEUROG1* expression, suggesting it might affect antitumor immune response via antigen presentation process. Nonetheless, we should acknowledge that the predictive performance of individual gene is very limited since SCLC often possesses the complex genomic and immune components.

Previously, the application of NMF to RNA‐seq data of 81 limited‐stage SCLC samples has identified four distinct subtypes with different immune features and therapeutic vulnerabilities [42, 43]. A recent study conducted de novo NMF using 271 ES‐SCLC patients from the IMpower133 and found patients with different treatment outcomes to atezolizumab plus carboplatin and etoposide [44]. Inspired by these results, we also performed de novo NMF to raw RNA‐seq data of 38 ES‐SCLC patients received camrelizumab plus nab‐paclitaxel and carboplatin based on the immune cell fractions. The results showed that the current set could also be divided into two well‐defined subsets with distinct immune features and clinical outcomes to first‐line chemo‐immunotherapy. These findings together suggest that deeper understanding of tumor immune microenvironment of SCLC by integrating multiomic data would be helpful to uncover the complex mechanisms underlying chemo‐immunotherapy responses.

Several limitations should be acknowledged. First, this study is limited by the sample size and hypothesis generating nature. Thus, the results of associations between genomic and transcriptomic features (such as *MUC17* alterations, *NEUROG1* expression level, TME1 and TME2) and response to camrelizumab plus nab‐paclitaxel and carboplatin need to be cautiously interpreted and will need further validation in larger randomized trials or an independent cohort. Second, this trial was a single‐arm, Phase 2 trial and all patients are Asians and recruited from a single regional hospital. No standard control arm was used. The selection bias is inevitable due to small sample size. The efficacy and safety profiles of camrelizumab plus carboplatin and nab‐paclitaxel in other regional hospitals remain to be confirmed. Third, the dose and schedule of chemotherapy might need further optimization. Administration of nab‐paclitaxel weekly could be further explored. Fourth, by the cutoff date, 27 (45%) patients had died. Longer follow‐up is needed to collect survival data. Last but not least, previous studies have divided SCLC into for molecular subtypes according to the expression of three significant transcription factors including ASCL1, NEUROD1, and POU2F3 [45]. Gay et al. further reported a classification for four subtypes with different response to first‐line chemo‐immunotherapy [43]. In this study, we did not analyze the associations between these predefined subtypes and response to camrelizumab plus carboplatin and nab‐paclitaxel mainly due to limited sample size. Instead, we performed de novo NMF and found similar findings.

In summary, this Phase 2 trial firstly provides evidence for the efficacy, safety, and potential predictors of camrelizumab plus carboplatin and nab‐paclitaxel as first‐line setting in ES‐SCLC, suggesting that this regimen could serve as an alternative first‐line setting for these patients. Meanwhile, integration of multiomic data to unveil the features of tumor immune microenvironment of SCLC would be helpful to clarify the complex mechanisms underlying chemo‐immunotherapy responses.

## 4 Methods

### 4.1 Study Design

This study was a single‐arm Phase 2 trial. Eligible criteria were (i) 18–75 years old, (ii) cytologically or histologically confirmed ES‐SCLC according to Veterans Administration Lung Study Group staging system, (iii) without previously antitumor treatment, (iv) ECOG performance status score of 0 or 1, (v) had measurable disease according to Response Evaluation Criteria in Solid Tumors version 1.1, (vi) life expectancy ≥ 3 months, and (vii) adequate organ function. Patients with asymptomatic CNS metastases, limited to the supratentorial region and cerebellum metastases were eligible. Key exclusion criteria included active serious infections, active or previous autoimmune diseases, and corticosteroid use within 2 weeks before the first study dose. The study protocol was reviewed and approved by the Ethics Committee of our hospital (approval no. L20‐446‐1). All patients provided written informed consent before enrollment. The trial was conducted in compliance with the Declaration of Helsinki and Good Clinical Practice guidelines, and has been registered in ClinicalTrial.gov (identifier: NCT04790539).

### 4.2 Procedures

Patients were given four to six cycles of nab‐paclitaxel (230 mg/m^2^ of body‐surface area on day 1 of a 3‐week cycle) and carboplatin (area under the curve of 5 mg/mL/min on day 1 of a 3‐week cycle) with camrelizumab (200 mg on day 1 of a 3‐week cycle), followed by maintenance therapy with camrelizumab (200 mg on day 1 of a 3‐week cycle). As per local standard practice, patients could receive prophylactic cranial irradiation during the maintenance phase, but thoracic radiotherapy in not permitted. Treatment would stop if PD, investigator decision, patient withdrawal, unacceptable toxicity, or up to 2 years of treatment with camrelizumab occurred. Dose interruptions of camrelizumab were permitted for but dose modification of camrelizumab was not allowed.

### 4.3 Endpoints and Response Assessments

The primary endpoint was 6‐month PFS rate (rate of patients who are alive and progression free at 6 months from first study dose). Secondary endpoints included ORR (proportion of patients with confirmed RECIST‐defined CR or PR), DCR (proportion of patients with RECIST‐defined CR, PR or SD lasting for ≥ 4 weeks), PFS (time from first study dose to RECIST‐defined PD or death, whichever occurred first), OS (time from first study dose to death), and safety. Investigator performed the tumor response assessment according to RECIST v1.1 every 6 weeks until Week 48, and then every 9 weeks until PD. CR and PR need to be confirmed at least 4 weeks after the first record. OS was recorded every 30 days during follow‐up. Investigators recorded and graded AEs according to National Cancer Institute Common Terminology Criteria for Adverse Events v5.0. AEs and SAEs were monitored from the time of informed consent to 90 days after the last camrelizumab treatment.

### 4.4 Sample Collection and Preparation

We collected the pretreatment tumor biopsy tissues and matched peripheral blood (10 mL). Genomic DNA and RNA from tumor samples were extracted using QIAamp DNA/RNA FFPE Tissue Kit and DNA from peripheral blood samples were extracted using DNeasy Blood Kit (Qiagen, GmBH, Germany). Library preparations were performed using KAPA HyperPrep Kit (KAPA Biosystems, Wilmington, MA, USA).

### 4.5 Whole‐Exome Sequencing

Whole‐exome capture was conducted using SureSelect Human All Exon Version 6.0 kit on tumor tissues and peripheral blood mononuclear cell (PBMC) samples. xGen universal blocking oligos (Integrated DNA Technologies) and Human Cot‐1 DNA (Life Technologies) were added to eliminate nonspecific hybridization. NimbleGen SeqCap EZ Hybridization (Life Technologies), Wash Kit, and Dynabeads M‐270 (Life Technologies) were used to conduct the capture reaction per manufacturers’ protocols. The captured high‐quality samples were sequenced on the Illumina HiSeq4000 platform using PE150 sequencing chemistry. The generated FASTQ files were converted to a BAM file for subsequent analyses.

### 4.6 Whole‐Exome Sequencing Data Analysis

We conducted the FastQC for quality control on the raw sequence data. Fastp was used for data cleaning [46]. To conduct a thorough analysis of whole‐exome sequencing (WES) data, we initiated with the identification and filtration of somatic single nucleotide variants (SNVs). This was achieved through the GATK [47, 48, 49]. Given the absence of paired normal samples, we employed a public panel of normal to distinguish somatic mutations from germline variants. Subsequently, we leveraged the “maftools” R package to scrutinize the list of detected mutations for biological significance [50]. This step was crucial given the reliance on a public normal panel, which necessitates additional filtering to prioritize mutations with potential clinical relevance. Our analysis culminated in the identification of the top 20 most mutated genes. Focusing on the top 20 most altered genes, we stratified the samples into two distinct subgroups based on the presence or absence of mutations in these genes or gene pairs in turn. This stratification aimed to discern any association between specific mutations and clinical outcomes. To statistically evaluate the effect of these mutations on clinical outcomes, we calculated *p* values using the log‐rank test. We reported cases where at least one of the *p* values from the log‐rank test for PFS or OS was less than 0.05.

### 4.7 Whole Transcriptome Sequencing

The capillary electrophoresis on a Eukaryote Total RNA Pico chip (Agilent Technologies) was performed to test RNA quality and quantity. The SMART‐Seq version 4 Ultra Low Input RNA Kit was used to prepare and amplify cDNA from 500 pg of RNA per manufacturer's instructions. The capillary electrophoresis on a Bioanalyzer using High Sensitivity chips (Agilent Technologies) was performed to determine the quality of amplified cDNA. The prepared libraries were also sequenced on Illumina HiSeq4000 platform using PE150 sequencing chemistry.

### 4.8 Whole Transcriptome Sequencing Data Analysis

Raw sequence reads were aligned to the hg19 using GSNAP. Then, mapped reads were assigned to human genes based on GRCh37.75 annotation. Uniquely mapped reads were used to quantify gene expression. A subpackage of Cufflinks v2.1.1 with Ensembl‐annotated genes v77 was used to analyze the DEGs. The abundance of transcripts was calculated and normalized in fragments per kilobase of transcript per million mapped reads. To delve deeper into the molecular differences between patient subgroups, we embarked on a differential expression analysis aimed at pinpointing genes that were dramatically upregulated or downregulated utilizing the “DESeq2” R package. The stringent criteria for identifying DEGs were set with an absolute fold change greater than 2, indicating a substantial change in gene expression levels between the subgroups. In addition, we applied a Benjamini–Hochberg‐corrected *p* value threshold of 0.05 to check the false discovery rate (FDR), ensuring that the identified DEGs were statistically robust. Upon identifying the DEGs, we proceeded to uncover the biological functions and pathways that these genes were associated with. To this end, we performed a comprehensive functional enrichment analysis of GO terms. This analysis was performed using the “clusterProfiler” R package, which enabled us to map the DEGs to GO terms and assess their statistical overrepresentation in the context of the entire genome.

### 4.9 Characterization of Immune Infiltration

To elucidate the landscape of immune infiltration, we employed the CIBERSORT algorithm. The “IOBR” R package facilitated the application of CIBERSORT, enabling us to deconvolute the complex mixture of tumor‐infiltrating lymphocytes present within the tumor immune microenvironment. Our analysis encompassed a comprehensive set of 22 distinct immune cell types. The significance of the differences in immune cell composition across patient subgroups was rigorously assessed using the *t*‐test. To visually represent the patterns of immune cell infiltration and to identify clusters of patients with similar immune profiles, we applied hierarchical clustering to the CIBERSORT output. This unsupervised method, also implemented through the “IOBR” R package, grouped samples based on their immune cell composition, revealing inherent structures within the data that were not apparent through other means. The clustering dendrogram and heatmap provided a synoptic view of the immune landscape.

### 4.10 Statistical Analysis

A one‐sided, one‐sample log‐rank test calculated from a sample of 54 subjects achieves 80.7% power at a 0.025 significance level to detect a 6‐month PFS rate of 35% in new group when the historic data is 20%. The investigators hypothesized a 10% dropout rate. The total sample size was 60. The ORR and DCR, and the corresponding 95% confidence intervals (CIs) were calculated using the Clopper– Pearson method. The PFS, OS and DoR, and the 95% CIs were calculated with the Brookmeyer and Crowley method by using Kaplan–Meier method. The SAS software version 9.4 (SAS Institute Inc. Cary, NC, USA) was used to conduct all statistical analyses. Two‐side *p* < 0.05 was considered as statistically significant.

## Author Contributions

Conception and design: S.R., T.J., and X.C. Administrative support: J. Yu, S.R., and C. Zhou. Provision of study materials or patients: S.R., J. Yu, S.Z., X.Z., A.X., Q.W., L.W., B.C., M.Y., W.L., and Y.W. Collection and assembly of data: S.R., J. Yu, J. Yuan, C. Zhao, and W.S. Data analysis and interpretation: S.R., T.J., X.C., L.C., D.C., J. Yuan, and C. Zhao. Manuscript writing: all authors. All authors have read and approved the final manuscript.

## Ethics Statement

This trial was approved by the Ethics Committee of Shanghai Pulmonary Hospital (approval no. L20‐446‐1), and conducted in accordance with the Declaration of Helsinki. All participants provided written informed consent upon enrollment.

## Conflicts of Interest

Shengxiang Ren has reported receiving speaker honoraria from Roche, Lilly, Boehringer Ingelheim, Hengrui, Merck Sharp & Dohme, and Junshi, and advisory fees from Hengrui, Merck Sharp & Dohme, and Roche. No further disclosures were reported. The other authors declare no conflicts of interest.

## Supporting information



Supporting Information

## Data Availability

The raw sequencing data have been uploaded online and are available via this link: https://doi.org/10.6084/m9.figshare.25512991.v1. Other data are available from the corresponding author upon reasonable request.

## References

[1] K. L. Simpson , D. G. Rothwell , F. Blackhall , and C. Dive , “Challenges of Small Cell Lung Cancer Heterogeneity and Phenotypic Plasticity,” Nature Reviews Cancer 25, no. 6 (2025): 447–462. 40211072 10.1038/s41568-025-00803-0

[2] T. Sen , N. Takahashi , S. Chakraborty , et al., “Emerging Advances in Defining the Molecular and Therapeutic Landscape of Small‐Cell Lung Cancer,” Nature Reviews Clinical Oncology 21, no. 8 (2024): 610–627. 10.1038/s41571-024-00914-x PMC11875021 38965396

[3] H. Sung , J. Ferlay , R. L. Siegel , et al., “Global Cancer Statistics 2020: Globocan Estimates of Incidence and Mortality Worldwide for 36 Cancers in 185 Countries,” CA: A Cancer Journal for Clinicians 71, no. 3 (2021): 209–249. 33538338 10.3322/caac.21660

[4] J. P. van Meerbeeck , D. A. Fennell , and D. K. De Ruysscher , “Small‐Cell Lung Cancer,” Lancet 378, no. 9804 (2011): 1741–1755. 21565397 10.1016/S0140-6736(11)60165-7

[5] J. K. Sabari , B. H. Lok , J. H. Laird , J. T. Poirier , and C. M. Rudin , “Unravelling the Biology of SCLC: Implications for Therapy,” Nature Reviews Clinical Oncology 14, no. 9 (2017): 549–561. 10.1038/nrclinonc.2017.71 PMC5843484 28534531

[6] L. E. Gaspar , E. J. McNamara , E. G. Gay , et al., “Small‐Cell Lung Cancer: Prognostic Factors and Changing Treatment Over 15 Years,” Clinical Lung Cancer 13, no. 2 (2012): 115–122. 22000695 10.1016/j.cllc.2011.05.008

[7] S. Wang , J. Tang , T. Sun , et al., “Survival Changes in Patients With Small Cell Lung Cancer and Disparities Between Different Sexes, Socioeconomic Statuses and Ages,” Scientific Reports 7, no. 1 (2017): 1339. 28465554 10.1038/s41598-017-01571-0 PMC5431017

[8] A. Schwendenwein , Z. Megyesfalvi , N. Barany , et al., “Molecular Profiles of Small Cell Lung Cancer Subtypes: Therapeutic Implications,” Molecular Therapy Oncolytics 20 (2021): 470–483. 33718595 10.1016/j.omto.2021.02.004 PMC7917449

[9] A. F. Farago and F. K. Keane , “Current Standards for Clinical Management of Small Cell Lung Cancer,” Translational Lung Cancer Research 7, no. 1 (2018): 69–79. 29535913 10.21037/tlcr.2018.01.16 PMC5835595

[10] G. S. Jones , K. Elimian , D. R. Baldwin , R. B. Hubbard , and T. M. McKeever , “A Systematic Review of Survival Following Anti‐Cancer Treatment for Small Cell Lung Cancer,” Lung Cancer 141 (2020): 44–55. 31955000 10.1016/j.lungcan.2019.12.015

[11] I. K. Demedts , K. Y. Vermaelen , and J. P. van Meerbeeck , “Treatment of Extensive‐Stage Small Cell Lung Carcinoma: Current Status and Future Prospects,” European Respiratory Journal 35, no. 1 (2010): 202–215. 20044461 10.1183/09031936.00105009

[12] N. Hanna , P. A. Bunn Jr. , C. Langer , et al., “Randomized Phase III Trial Comparing Irinotecan/Cisplatin With Etoposide/Cisplatin in Patients With Previously Untreated Extensive‐Stage Disease Small‐Cell Lung Cancer,” Journal of Clinical Oncology 24, no. 13 (2006): 2038–2043. 16648503 10.1200/JCO.2005.04.8595

[13] K. Noda , Y. Nishiwaki , M. Kawahara , et al., “Irinotecan Plus Cisplatin Compared With Etoposide Plus Cisplatin for Extensive Small‐Cell Lung Cancer,” New England Journal of Medicine 346, no. 2 (2002): 85–91. 11784874 10.1056/NEJMoa003034

[14] S. V. Liu , M. Reck , A. S. Mansfield , et al., “Updated Overall Survival and PD‐L1 Subgroup Analysis of Patients With Extensive‐Stage Small‐Cell Lung Cancer Treated With Atezolizumab, Carboplatin, and Etoposide (IMpower133),” Journal of Clinical Oncology 39, no. 6 (2021): 619–630. 33439693 10.1200/JCO.20.01055 PMC8078320

[15] J. W. Goldman , M. Dvorkin , Y. Chen , et al., “Durvalumab, With or Without Tremelimumab, Plus Platinum‐Etoposide Versus Platinum‐Etoposide Alone in First‐Line Treatment of Extensive‐Stage Small‐Cell Lung Cancer (Caspian): Updated Results From a Randomised, Controlled, Open‐Label, Phase 3 Trial,” Lancet Oncology 22, no. 1 (2021): 51–65. 33285097 10.1016/S1470-2045(20)30539-8

[16] J. Wang , C. Zhou , W. Yao , et al., “Adebrelimab or Placebo Plus Carboplatin and Etoposide as First‐Line Treatment for Extensive‐Stage Small‐Cell Lung Cancer (Capstone‐1): A Multicentre, Randomised, Double‐Blind, Placebo‐Controlled, Phase 3 Trial,” Lancet Oncology 23, no. 6 (2022): 739–747. 35576956 10.1016/S1470-2045(22)00224-8

[17] C. M. Rudin , M. M. Awad , A. Navarro , et al., “Pembrolizumab or Placebo Plus Etoposide and Platinum as First‐Line Therapy for Extensive‐Stage Small‐Cell Lung Cancer: Randomized, Double‐Blind, Phase III Keynote‐604 Study,” Journal of Clinical Oncology 38, no. 21 (2020): 2369–2379. 32468956 10.1200/JCO.20.00793 PMC7474472

[18] Y. Cheng , L. Han , L. Wu , et al., “Effect of First‐Line Serplulimab vs Placebo Added to Chemotherapy on Survival in Patients With Extensive‐Stage Small Cell Lung Cancer: The Astrum‐005 Randomized Clinical Trial,” JAMA 328, no. 12 (2022): 1223–1232. 36166026 10.1001/jama.2022.16464 PMC9516323

[19] Y. Cheng , Y. Fan , Y. Zhao , et al., “Tislelizumab Plus Platinum and Etoposide Versus Placebo Plus Platinum and Etoposide as First‐Line Treatment for Extensive‐Stage SCLC (RATIONALE‐312): A Multicenter, Double‐Blind, Placebo‐Controlled, Randomized, Phase 3 Clinical Trial,” Journal of Thoracic Oncology 19, no. 7 (2024): 1073–1085. 38460751 10.1016/j.jtho.2024.03.008

[20] Y. Cheng , W. Zhang , L. Wu , et al., “Toripalimab Plus Chemotherapy as a First‐Line Therapy for Extensive‐Stage Small Cell Lung Cancer: The Phase 3 EXTENTORCH Randomized Clinical Trial,” JAMA Oncology 11, no. 1 (2025): 16–25. 39541202 10.1001/jamaoncol.2024.5019 PMC11565370

[21] D. Adrianzen Herrera , N. Ashai , R. Perez‐Soler , and H. Cheng , “Nanoparticle Albumin Bound‐Paclitaxel for Treatment of Advanced Non‐Small Cell Lung Cancer: An Evaluation of the Clinical Evidence,” Expert Opinion on Pharmacotherapy 20, no. 1 (2019): 95–102. 30439289 10.1080/14656566.2018.1546290

[22] H. West , M. McCleod , M. Hussein , et al., “Atezolizumab in Combination With Carboplatin Plus Nab‐Paclitaxel Chemotherapy Compared With Chemotherapy Alone as First‐Line Treatment for Metastatic Non‐Squamous Non‐Small‐Cell Lung Cancer (IMpower130): A Multicentre, Randomised, Open‐Label, Phase 3 Trial,” Lancet Oncology 20, no. 7 (2019): 924–937. 31122901 10.1016/S1470-2045(19)30167-6

[23] P. Schmid , S. Adams , H. S. Rugo , et al., “Atezolizumab and Nab‐Paclitaxel in Advanced Triple‐Negative Breast Cancer,” New England Journal of Medicine 379, no. 22 (2018): 2108–2121. 30345906 10.1056/NEJMoa1809615

[24] S. Ren , G. Gao , W. Li , et al., “P1.15‐009 Safety and Efficacy of Nab‐Paclitaxel Monotherapy as 2nd or Later Line Setting in Pts With Extensive SCLC, a Phase II Single Arm Study (Nct02262897),” Journal of Thoracic Oncology 12, no. 11 (2017): S2046.

[25] Z. Megyesfalvi , C. M. Gay , H. Popper , et al., “Clinical Insights Into Small Cell Lung Cancer: Tumor Heterogeneity, Diagnosis, Therapy, and Future Directions,” CA: A Cancer Journal for Clinicians 73, no. 6 (2023): 620–652. 37329269 10.3322/caac.21785

[26] A. Solta , B. Ernhofer , K. Boettiger , et al., “Small Cells—Big Issues: Biological Implications and Preclinical Advancements in Small Cell Lung Cancer,” Molecular Cancer 23, no. 1 (2024): 41. 38395864 10.1186/s12943-024-01953-9 PMC10893629

[27] C. M. Rudin , E. Brambilla , C. Faivre‐Finn , and J. Sage , “Small‐Cell Lung Cancer,” Nature Reviews Disease Primers 7, no. 1 (2021): 3. 10.1038/s41572-020-00235-0 PMC8177722 33446664

[28] S. Ren , J. Chen , X. Xu , et al., “Camrelizumab Plus Carboplatin and Paclitaxel as First‐Line Treatment for Advanced Squamous NSCLC (Camel‐Sq): A Phase 3 Trial,” Journal of Thoracic Oncology 17, no. 4 (2022): 544–557. 34923163 10.1016/j.jtho.2021.11.018

[29] L. Paz‐Ares , A. Luft , D. Vicente , et al., “Pembrolizumab Plus Chemotherapy for Squamous Non‐Small‐Cell Lung Cancer,” New England Journal of Medicine 379, no. 21 (2018): 2040–2051. 30280635 10.1056/NEJMoa1810865

[30] P. Thomas , O. Castelnau , D. Paillotin , et al., “Phase II Trial of Paclitaxel and Carboplatin in Metastatic Small‐Cell Lung Cancer: A Groupe Français De Pneumo‐Cancérologie Study,” Journal of Clinical Oncology 19, no. 5 (2001): 1320–1325. 11230474 10.1200/JCO.2001.19.5.1320

[31] C. Gridelli , L. Manzione , F. Perrone , et al., “Carboplatin Plus Paclitaxel in Extensive Small Cell Lung Cancer: A Multicentre Phase 2 Study,” British Journal of Cancer 84, no. 1 (2001): 38–41. 11139310 10.1054/bjoc.2000.1541 PMC2363602

[32] J. D. Lickliter , H. K. Gan , M. Voskoboynik , et al., “A First‐in‐Human Dose Finding Study of Camrelizumab in Patients With Advanced or Metastatic Cancer in Australia,” Drug Design, Development and Therapy 14 (2020): 1177–1189. 32256049 10.2147/DDDT.S243787 PMC7090185

[33] M. Untch , C. Jackisch , A. Schneeweiss , et al., “Nab‐Paclitaxel Versus Solvent‐Based Paclitaxel in Neoadjuvant Chemotherapy for Early Breast Cancer (GeparSepto‐GBG 69): A Randomised, Phase 3 Trial,” Lancet Oncology 17, no. 3 (2016): 345–356. 26869049 10.1016/S1470-2045(15)00542-2

[34] N. Saijo , “The Role of Pharmacoethnicity in the Development of Cytotoxic and Molecular Targeted Drugs in Oncology,” Yonsei Medical Journal 54, no. 1 (2013): 1–14. 23225792 10.3349/ymj.2013.54.1.1 PMC3521281

[35] L. Horn , A. S. Mansfield , A. Szczęsna , et al., “First‐Line Atezolizumab Plus Chemotherapy in Extensive‐Stage Small‐Cell Lung Cancer,” New England Journal of Medicine 379, no. 23 (2018): 2220–2229. 30280641 10.1056/NEJMoa1809064

[36] S. Lin , H. Ruan , L. Qin , et al., “Acquired Resistance to EGFR‐TKIs in NSCLC Mediates Epigenetic Downregulation of MUC17 by Facilitating NF‐κB Activity via UHRF1/DNMT1 Complex,” International Journal of Biological Sciences 19, no. 3 (2023): 832–851. 36778111 10.7150/ijbs.75963 PMC9910003

[37] W. S. Al Amri , L. M. Allinson , D. E. Baxter , et al., “Genomic and Expression Analyses Define MUC17 and PCNX1 as Predictors of Chemotherapy Response in Breast Cancer,” Molecular Cancer Therapeutics 19, no. 3 (2020): 945–955. 31879365 10.1158/1535-7163.MCT-19-0940

[38] B. Yang , A. Wu , Y. Hu , et al., “Mucin 17 Inhibits the Progression of Human Gastric Cancer by Limiting Inflammatory Responses Through a MYH9‐p53‐RhoA Regulatory Feedback Loop,” Journal of Experimental & Clinical Cancer Research 38, no. 1 (2019): 283. 31262330 10.1186/s13046-019-1279-8 PMC6604468

[39] C. P. Wardell , M. Fujita , T. Yamada , et al., “Genomic Characterization of Biliary Tract Cancers Identifies Driver Genes and Predisposing Mutations,” Journal of Hepatology 68, no. 5 (2018): 959–969. 29360550 10.1016/j.jhep.2018.01.009

[40] G. C. Machado and V. P. Ferrer , “MUC17 Mutations and Methylation Are Associated With Poor Prognosis in Adult‐Type Diffuse Glioma Patients,” Journal of the Neurological Sciences 452 (2023): 120762. 37562166 10.1016/j.jns.2023.120762

[41] A. Herbst , K. Rahmig , P. Stieber , et al., “Methylation of NEUROG1 in Serum Is a Sensitive Marker for the Detection of Early Colorectal Cancer,” American Journal of Gastroenterology 106, no. 6 (2011): 1110–1118. 21326223 10.1038/ajg.2011.6

[42] J. George , J. S. Lim , S. J. Jang , et al., “Comprehensive Genomic Profiles of Small Cell Lung Cancer,” Nature 524, no. 7563 (2015): 47–53. 26168399 10.1038/nature14664 PMC4861069

[43] C. M. Gay , C. A. Stewart , E. M. Park , et al., “Patterns of Transcription Factor Programs and Immune Pathway Activation Define Four Major Subtypes of SCLC With Distinct Therapeutic Vulnerabilities,” Cancer Cell 39, no. 3 (2021): 346–360. 33482121 10.1016/j.ccell.2020.12.014 PMC8143037

[44] B. Y. Nabet , H. Hamidi , M. C. Lee , et al., “Immune Heterogeneity in Small‐Cell Lung Cancer and Vulnerability to Immune Checkpoint Blockade,” Cancer Cell 42, no. 3 (2024): 429–443.e4. 38366589 10.1016/j.ccell.2024.01.010

[45] C. M. Rudin , J. T. Poirier , L. A. Byers , et al., “Molecular Subtypes of Small Cell Lung Cancer: A Synthesis of Human and Mouse Model Data,” Nature Reviews Cancer 19, no. 5 (2019): 289–297. 30926931 10.1038/s41568-019-0133-9 PMC6538259

[46] S. Chen , Y. Zhou , Y. Chen , and J. Gu , “Fastp: An Ultra‐Fast All‐in‐One FASTQ Preprocessor,” Bioinformatics 34, no. 17 (2018): i884–i890. 30423086 10.1093/bioinformatics/bty560 PMC6129281

[47] H. Li , B. Handsaker , A. Wysoker , et al., “The Sequence Alignment/Map Format and SAMtools,” Bioinformatics 25, no. 16 (2009): 2078–2079. 19505943 10.1093/bioinformatics/btp352 PMC2723002

[48] A. McKenna , M. Hanna , E. Banks , et al., “The Genome Analysis Toolkit: A MapReduce Framework for Analyzing Next‐Generation DNA Sequencing Data,” Genome Research 20, no. 9 (2010): 1297–1303. 20644199 10.1101/gr.107524.110 PMC2928508

[49] M. A. DePristo , E. Banks , R. Poplin , et al., “A Framework for Variation Discovery and Genotyping Using Next‐Generation DNA Sequencing Data,” Nature Genetics 43, no. 5 (2011): 491–498. 21478889 10.1038/ng.806 PMC3083463

[50] A. Mayakonda , D.‐C. Lin , Y. Assenov , C. Plass , and H. P. Koeffler , “Maftools: Efficient and Comprehensive Analysis of Somatic Variants in Cancer,” Genome Research 28, no. 11 (2018): 1747–1756. 30341162 10.1101/gr.239244.118 PMC6211645

