# Supplementary material for: Camrelizumab, an Anti‐PD‐1 Monoclonal Antibody, Plus Carboplatin and Nab‐Paclitaxel as First‐Line Setting for Extensive‐Stage Small‐Cell Lung Cancer: A Phase 2 Trial and Biomarker Analysis
Source: MedComm (2020). 2025 Jul 27;6(8):e70300. doi: 10.1002/mco2.70300 (PMC12301171; doi:10.1002/mco2.70300)
Supplement: Supplementary file 1 — Supporting Information [file MCO2-6-e70300-s001.docx]

**Supplemental materials**

**Camrelizumab plus nab-paclitaxel and carboplatin as first-line treatment for extensive-stage small-cell lung cancer (ES-SCLC): a phase 2 trial and biomarker analysis**

**Supplemental Table S1………………………………………………………….…………2**

**Supplemental Table S2……………………………………………………………….……3**

**Supplemental Table S3………………………………………………………………...…..4**

**Supplemental Figure 1………………………………………………………………….…5**

**Supplemental Figure 2………………………………………………………………….…6**

**Supplemental Figure 3………………………………………………………………….…7**

**Supplemental Figure 4………………………………………………………………….…8**

**Supplemental Figure 5………………………………………………………….…………9**

**Supplemental Figure 6………………………………………………………………..…..10**

**Supplemental Figure 7…………………………………………………………………....11**

**Supplemental** **Table S1. Summary of objective response for all included patients.**

| **Variables** | **All patients (n=60)** |
| --- | --- |
| Best overall response, n (%) |  |
| Complete response (confirmed) | 1 (1.7) |
| Partial response (confirmed) | 43 (71.7) |
| Stable disease | 12 (20.0) |
| Progressive disease | 1 (1.7) |
| Not evaluable^*^ | 3 (5.0) |
| Confirmed ORR, n (%; 95% CI) | 44 (73.3; 60.3-83.9) |
| DCR, n (%; 95% CI) | 56 (93.3; 83.8-98.2) |
| TTR, months, median (range) | 1.4 (0.7-2.7) |
| DoR, months, median (95% CI) | 6.7 (5.1–8.2) |
| ^*^, 3 patients were not evaluable due to study discontinuation (patient’s decision). ORR, objective response rate; DCR, disease control rate; TTR, time to response; DoR, duration of response; CI, confidence interval. | |

**Supplemental** **Table S2. Summary of immune-related adverse events for all included patients.**

| **Adverse events** | **All patients (n=60)** | |
| --- | --- | --- |
|  | Any grade | Grade ≥3 |
| Any | 21 (35.0%) | 13 (21.7%) |
| Immune-mediated hepatitis | 3 (5.0%) | 3 (5.0%) |
| Platelet count decreased | 3 (5.0%) | 2 (3.3%) |
| Hepatic function abnormal | 2 (3.3%) | 1 (1.6%) |
| Immune-mediated nephritis | 1 (1.6%) | 1 (1.6%) |
| Immune-mediated cystitis | 1 (1.6%) | 1 (1.6%) |
| Febrile neutropenia | 1 (1.6%) | 1 (1.6%) |
| Diabetes mellitus | 1 (1.6%) | 1 (1.6%) |
| Urinary tract infection | 1 (1.6%) | 1 (1.6%) |
| Lung infection | 1 (1.6%) | 1 (1.6%) |
| Agranulocytosis | 1 (1.6%) | 1 (1.6%) |

**Supplemental** **Table S3. Baseline characteristics of included patients from the biomarker evaluable population and intention-to-treat cohort.**

| **Characteristics** | **ITT (n=60)** | **BEP (n=41)** | ***P* value** |
| --- | --- | --- | --- |
| Age, years, median (range) | 64.5 (38-74) | 62.0 (32-73) |  |
| <65 years, n (%) | 34 (56.7) | 23 (56.1) | 0.955 |
| ≥65 years, n (%) | 26 (43.3) | 18 (43.9) |  |
| Male, n (%) | 50 (83.3) | 35 (85.4) | 0.784 |
| ECOG performance status, n (%) |  |  |  |
| 0 | 0 (0) | 0 (0) | - |
| 1 | 60 (100) | 41 (100) |  |
| Disease stage, n (%) |  |  |  |
| III | 8 (13.3) | 7 (17.1) | 0.604 |
| IV | 52 (86.7) | 34 (82.9) |  |
| Smoking status, n (%) |  |  |  |
| Current or former smoker | 40 (66.7) | 27 (65.9) | 0.932 |
| Never smoked | 20 (33.3) | 14 (34.1) |  |
| Brain metastases, n (%) |  |  |  |
| Yes | 7 (11.7) | 4 (9.8) | 0.982 |
| No | 53 (88.3) | 37 (90.2) |  |
| Liver metastases, n (%) |  |  |  |
| Yes | 10 (16.7) | 7 (17.1) | 0.957 |
| No | 50 (83.3) | 34 (82.9) |  |
| Lactate dehydrogenase at enrolment |  |  |  |
| ≤ULN | 39 (65.0) | 30 (73.2) | 0.132 |
| >ULN | 21 (35.0) | 11 (26.8) |  |
| ECOG, Eastern Cooperative Oncology Group; ULN, upper normal limit. | | | |


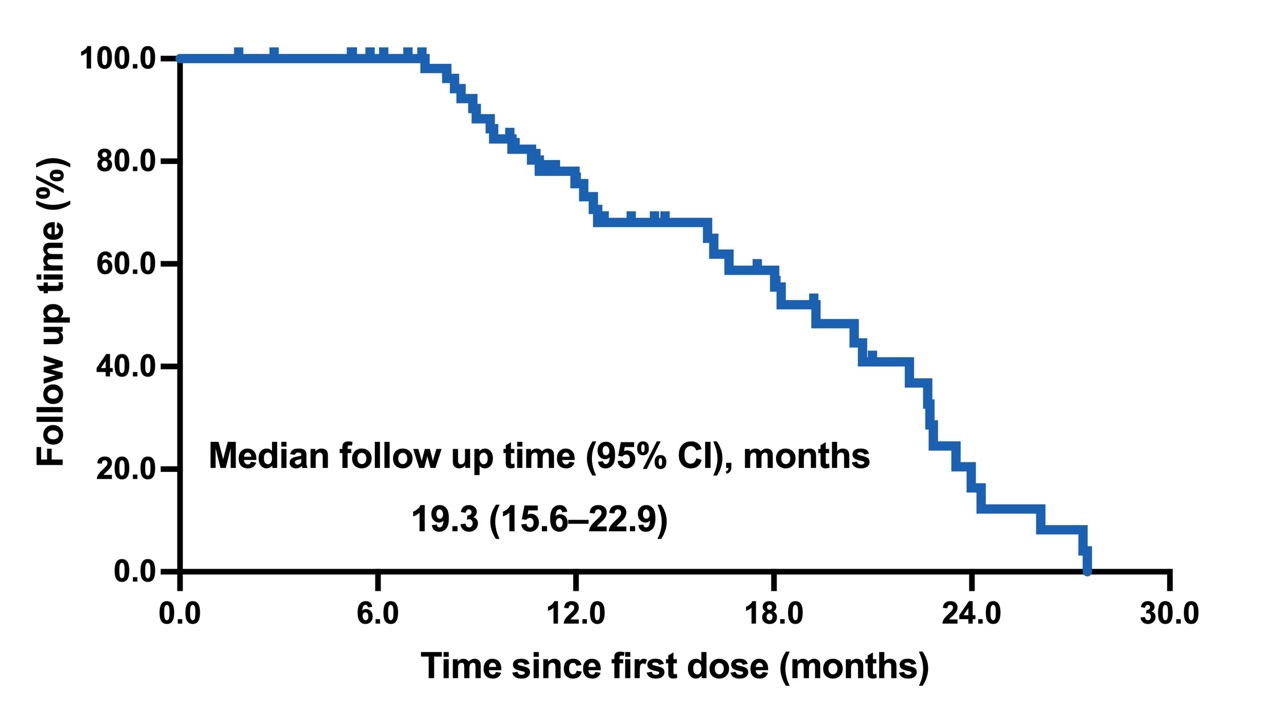


**Supplemental Figure 1. Kaplan-Meier curve for follow up time.**


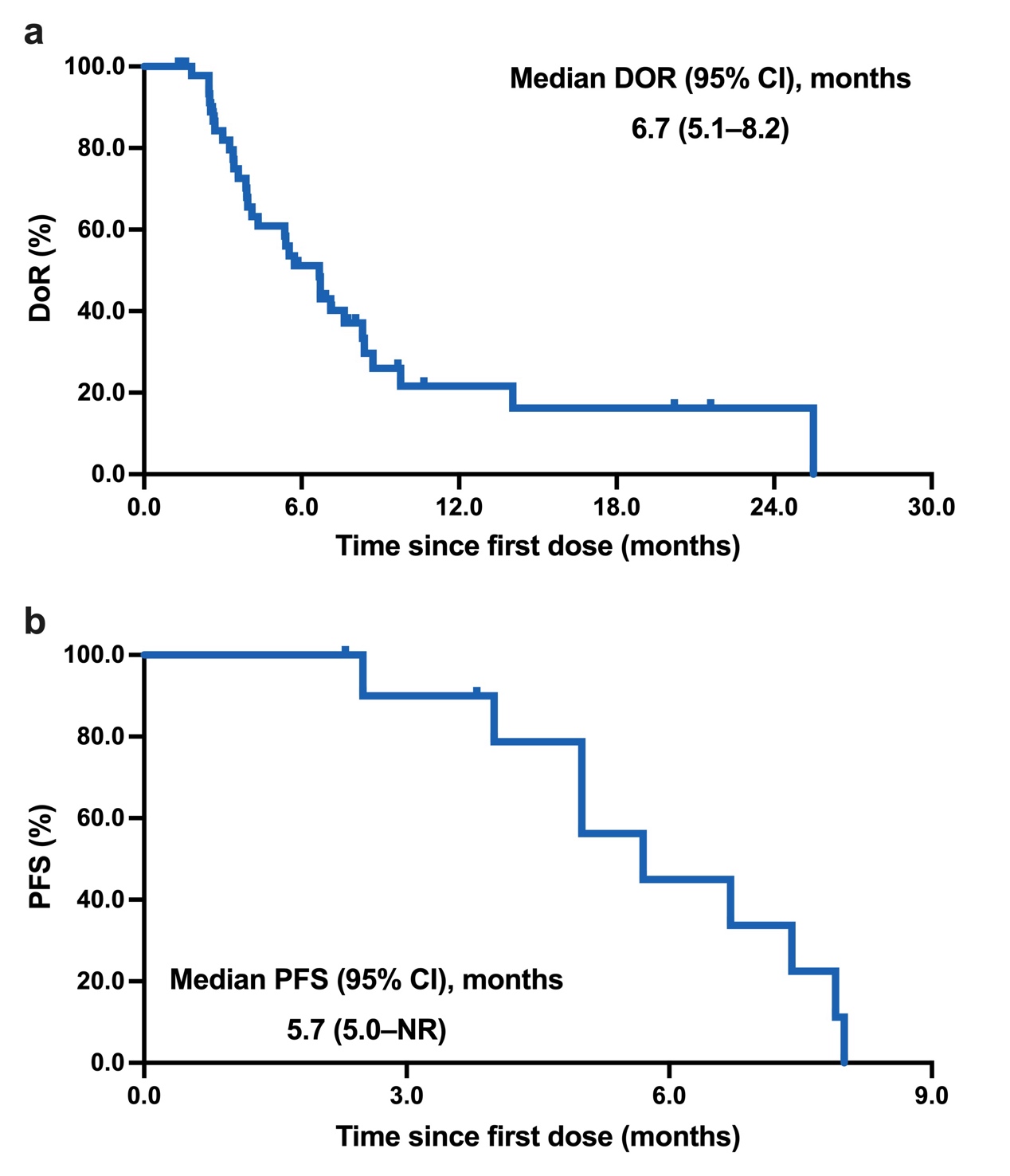


**Supplemental Figure 2. Kaplan-Meier curve for duration of response (a) and PFS of patients received platinum and etoposide as the second-line treatment after PD.** DoR, duration of response; PFS, progression-free survival; NR, not reached; CI, confidence interval.


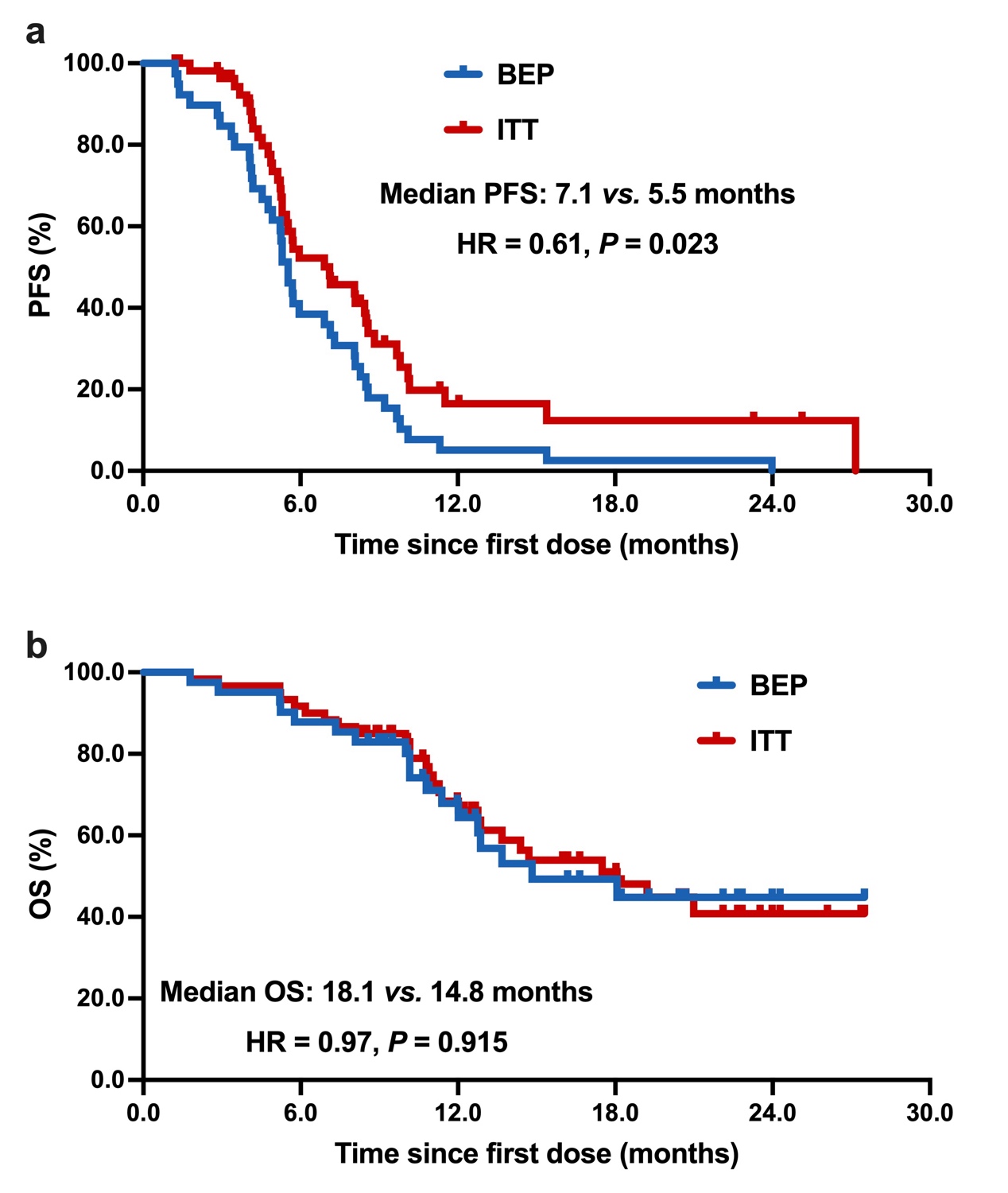


**Supplemental Figure 3. Clinical outcome comparison between biomarker-evaluable population (BEP) and intention-to-treat (ITT) population. a.** PFS comparison between BEP and ITT population. **b.** OS comparison between BEP and ITT population.

**
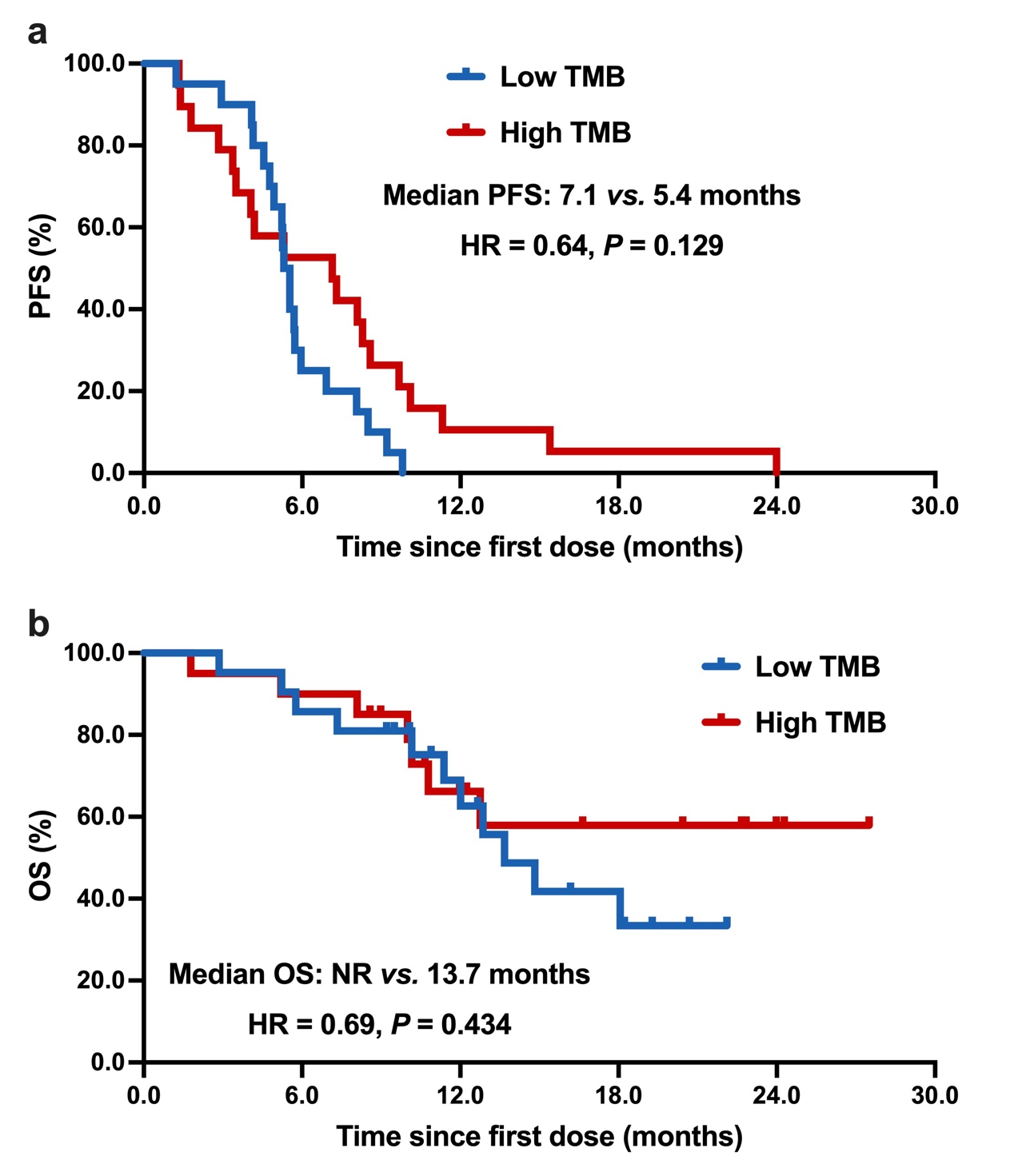
**

**Supplemental Figure 4. Clinical outcome comparison between patients with high and low TMB. a.** PFS comparison between patients with high and low TMB. **b.** OS comparison between patients with high and low TMB. PFS, progression-free survival; OS, overall survival; HR, hazard ratio; NR, not reached; TMB, tumor mutational burden.

**
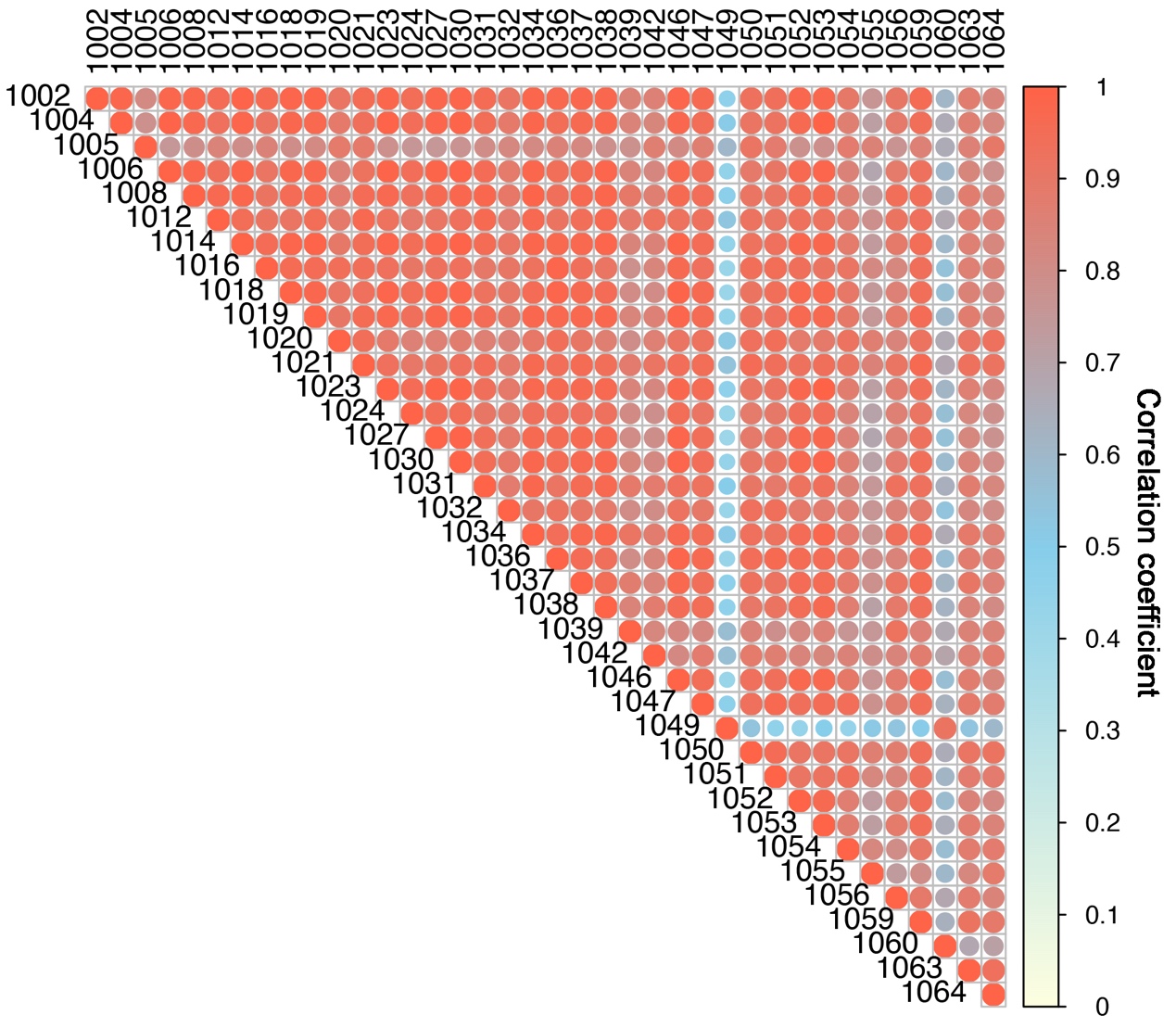
**

**Supplemental Figure 5. Sample correlation heat map based on expression matrix;**

**different colors represent different correlation coefficients.**

**
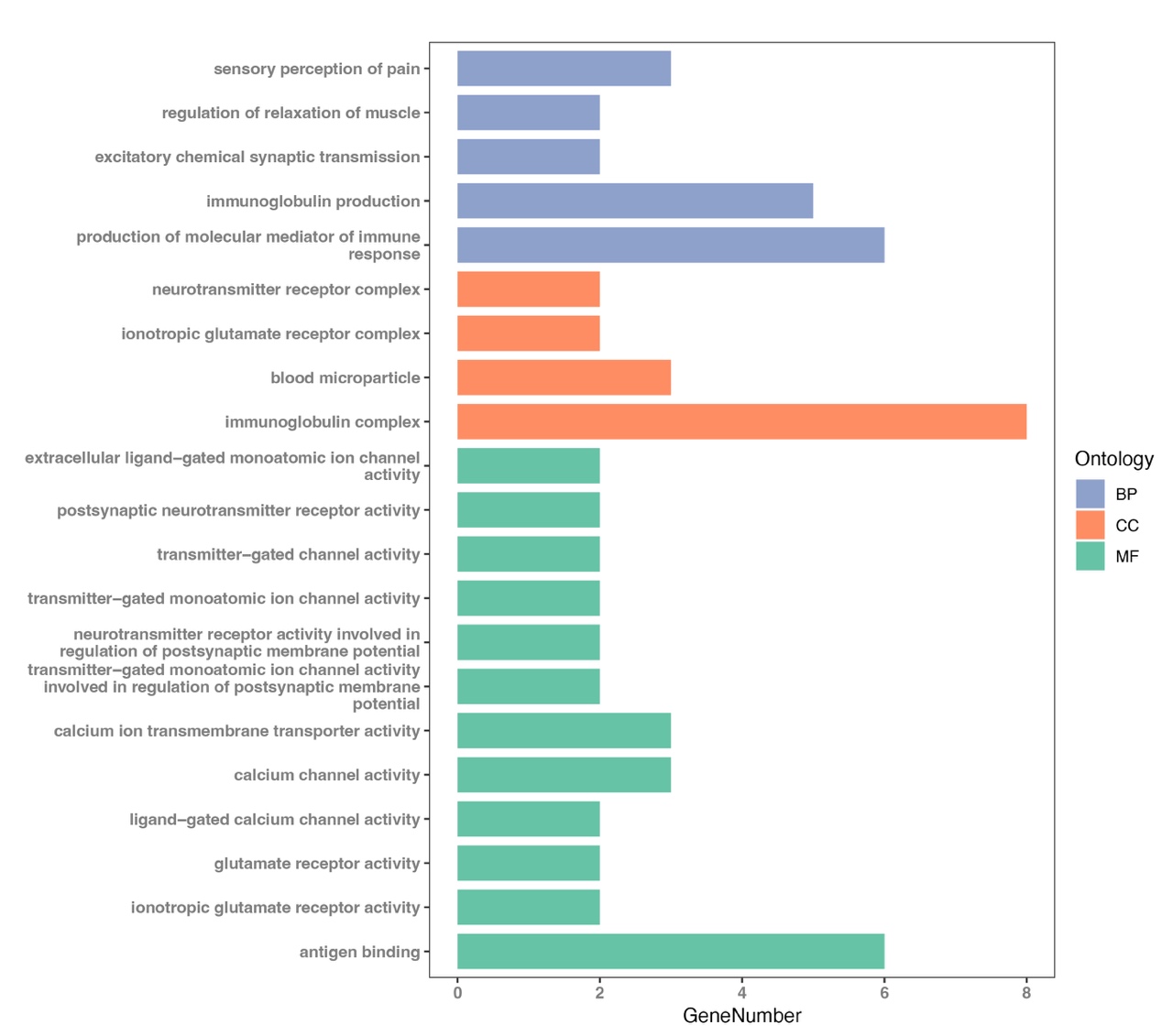
**

**Supplemental Figure 6. GO enrichment analyses showed that the differentially expressed genes between CR+PR and SD+PD groups prominently displayed immune-related functions including antigen binding and immunoglobulin production.**


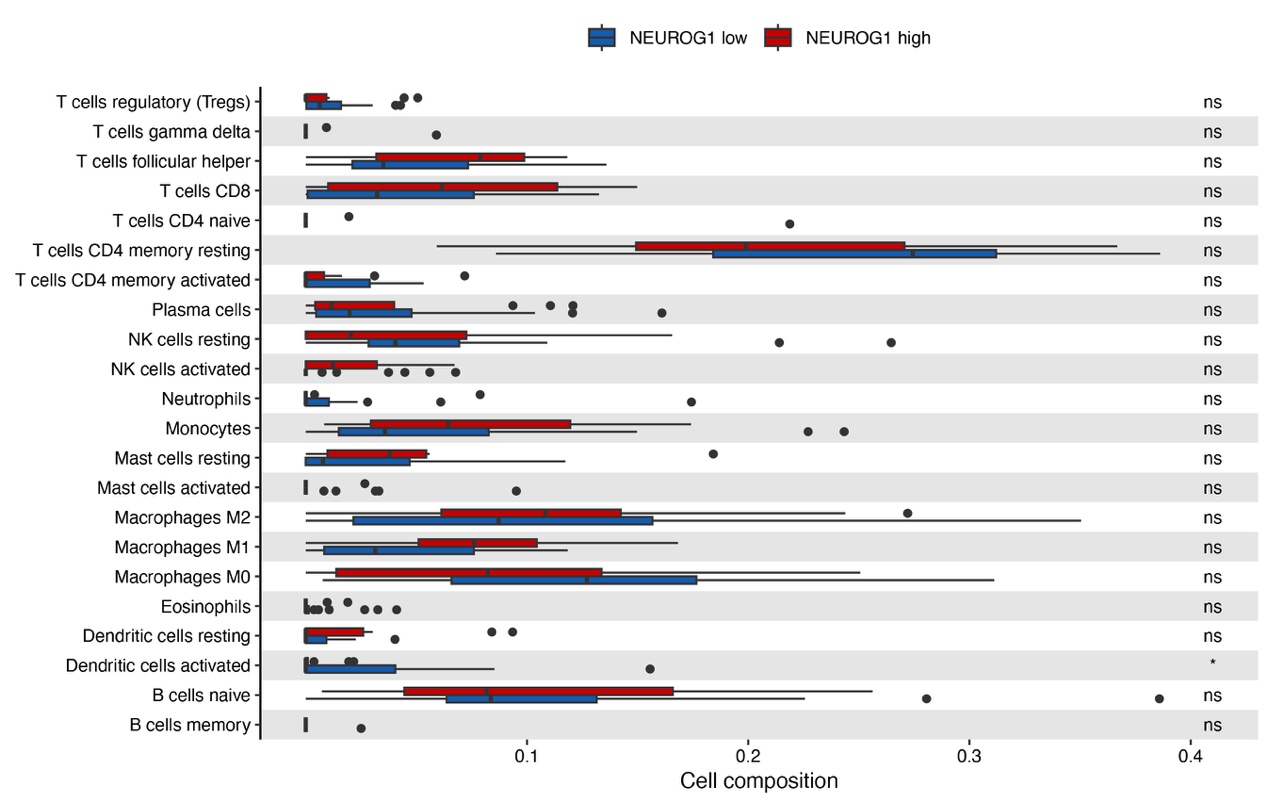


**Supplemental Figure 7.** **Comparison of immune infiltrates estimated by CIBERSORT between patients with high and low *NEUROG1* expression.**
